# Realizing better doctor-patient dialogue about choices in palliative care and early phase clinical trial participation: towards an online value clarification tool (OnVaCT)

**DOI:** 10.1186/s12904-019-0486-6

**Published:** 2019-11-29

**Authors:** Liza G. G. van Lent, Nicole K. Stoel, Julia C. M. van Weert, Jelle van Gurp, Maja J. A. de Jonge, Martijn P. Lolkema, Eelke H. Gort, Saskia M. Pulleman, Esther Oomen-de Hoop, Jeroen Hasselaar, Carin C. D. van der Rijt

**Affiliations:** 1 Department of Medical Oncology, Erasmus MC Cancer Institute, Rotterdam, the Netherlands; 2 Department of Anaesthesiology, Pain & Palliative Medicine, Radboud University Medical Centre, Nijmegen, the Netherlands; 3 Department of Communication Science, Amsterdam School of Communication Research (ASCoR) and University of Amsterdam, Amsterdam, the Netherlands; 4 Department of IQ Healthcare, Radboud University Medical Centre, Nijmegen, the Netherlands; 5 Department of Medical Oncology, UMC Utrecht Cancer Centre, Utrecht, the Netherlands; 6 Department of Medical Oncology and Clinical Pharmacology, Antoni van Leeuwenhoek, the Netherlands Cancer Institute, Amsterdam, the Netherlands

**Keywords:** Early phase clinical trial, eHealth, Ethics, Palliative care, Value clarification tool, Patient-physician communication, Patient-centred care, Shared decision making

## Abstract

**Background:**

Patients with advanced cancer for whom standard systemic treatment is no longer available may be offered participation in early phase clinical trials. In the decision making process, both medical-technical information and patient values and preferences are important. Since patients report decisional conflict after deciding on participation in these trials, improving the decision making process is essential. We aim to develop and evaluate an Online Value Clarification Tool (OnVaCT) to assist patients in clarifying their values around this end-of-life decision. This improved sharing of values is hypothesized to support medical oncologists in tailoring their information to individual patients’ needs and, consequently, to support patients in taking decisions in line with their values and reduce decisional conflict.

**Methods:**

In the first part, patients’ values and preferences and medical oncologists’ views hereupon will be explored in interviews and focus groups to build a first prototype OnVaCT using digital communication (serious gaming). Next, we will test feasibility during think aloud sessions, to deliver a ready-to-implement OnVaCT. In the second part, the OnVaCT, with accompanied training module, will be evaluated in a pre-test (12–18 months before implementation) post-test (12–18 months after implementation) study in three major Dutch cancer centres. We will include 276 patients (> 18 years) with advanced cancer for whom standard systemic therapy is no longer available, and who are referred for participation in early phase clinical trials. The first consultation will be recorded to analyse patient-physician communication regarding the discussion of patients’ values and the decision making process. Three weeks afterwards, decisional conflict will be measured.

**Discussion:**

This project aims to support the discussion of patient values when considering participation in early phase clinical trials. By including patients before their first appointment with the medical oncologist and recording that consultation, we are able to link decisional conflict to the decision making process, e.g. the communication during consultation. The study faces challenges such as timely including patients within the short period between referral and first consultation. Furthermore, with new treatments being developed rapidly, molecular stratification may affect the patient populations included in the pre-test and post-test periods.

**Trial registration:**

Netherlands Trial Registry number: NTR7551 (prospective; July 17, 2018).

## Background

Most patients with advanced cancer will ultimately reach the moment that standard anti-cancer therapy is no longer available. At that particular moment, some are still in a relatively good condition and may live for several months or longer. Those patients may be asked and/or considered to participate in early phase clinical trials. These trials are a major prerequisite for the further development of efficacious anti-cancer therapies. Despite improved molecular stratification for experimental therapy and thus improved anti-cancer activity in these clinical trials [1], the majority of patients do not benefit from participation. Although a recent search revealed a 19.8% response rate (both complete and partial responses) in phase I trials from 2014 until mid-2015 [2], it is known that participation can (also) be beneficial for other reasons. Hope for and belief in benefit are for instance important reasons for patients to participate [3, 4], and those factors can positively affect quality of life [5]. However, it is also known that patients can be aware of palliative care services at the end of life but may not consider them for themselves [6], which may especially apply to those who have unrealistic hope for benefit [4]. In that case, trial participation may interfere with adequate end-of-life decision making.

Deciding whether or not to participate in an early phase clinical trial thus is a complex decisional process. Decisional conflict, i.e. “the extent to which they [i.e. patients] report unresolved decisional needs such as personal uncertainty and related deficits in knowledge, values clarity, and support or pressure” [7, p69] has been found in patients after deciding on participation in an early phase clinical study [7], which seems to indicate that the current decision making process could be improved. Improving patients’ decision making process, independent from the ultimate content of the decision, has shown to be a way to improve health-related quality of life [8, 9]. Easing this major-impact decision therefore offers an opportunity to improve quality of life for these patients.

Important factors to optimize the decision making process are defining personal values and weighing the available information on the proposed interventions [10]. In the consent procedure preceding clinical trial participation, patients’ values and preferences together with more technical information need to be discussed. Systematic reviews and empirical research suggest that effective doctor-patient communication requires knowledge about values and preferences of patients [11, 12]. However, given the complexity of early phase clinical trials nowadays, focus on the information about the specific clinical trial (s) may prevail with a limited patient-physician discussion on patient values. Furthermore, information on actual prognosis and available palliative care options are not always given [13, 14]. Incomplete information provision may cause unmet communication needs [12], which supports the hypothesis that effective decision making for trial participation could benefit from more focus on patients values and preferences. However there is a lack of studies that investigate potential interventions to improve this aspect [12]. Improving patient-oncologist communication into a more patient-based and value-centred dialogue could potentially be achieved through focussing on true “human connection” [15].

Decision aids can offer support in this complex decision making process. Although not investigated in the context of participation in early phase clinical trials, the use of decision aids in other medical situations has been found to improve patient-provider communication and reduce decisional conflict [16]. However, decision aids do not particularly focus at the previously mentioned human connection. Eliciting patients’ values and preferences better reflects this connection and is a key part of the shared decision making process in widely used shared decision making models [17–19]. Therefore, according to the International Patient Decision Aid Standards (IPDAS), the inclusion of value clarification in decision aids is strongly recommended [20]. Indeed, exposure to a decision aid with explicit value clarification resulted in a higher proportion of patients choosing an option congruent with their values [16]. This suggests that singly clarifying one’s values could already improve the decision making process. In particular, value clarification needs to be attuned to a personal logic that is based on context in order not to be burdensome [21].

We will develop an online value clarification tool (OnVaCT) with accompanying training to help patients explore their own values and attitudes towards palliative care and treatment within an early phase clinical trial in order to incorporate this information in the patient-oncologist communication. We will evaluate the OnVaCT by assessing the communication process regarding the extent to which caregivers involve patients in the decision making process and the patients’ decisional conflict. We hypothesize that clarifying patients’ values in preparing for the first consultation with a medical oncologist (i.e. the consultation that sets the consent procedure in motion) will help patients to more easily share their personal values with their medical oncologist. In turn, these insights may assist oncologists in aligning the information offered with the values considered important by patients. Ultimately, we hypothesize that this intervention will reduce decisional conflict.

## Methods

### Overall aim

**Fig. 1:**
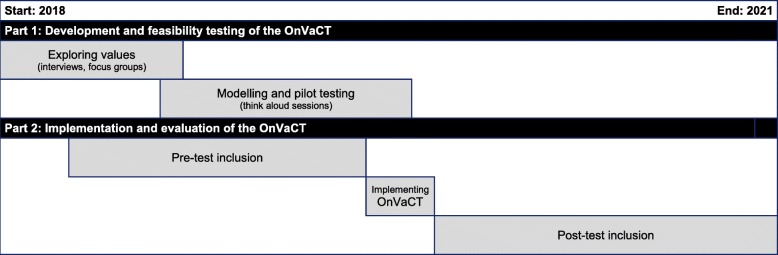
Schematic overview of the OnVaCT project

The overall aim of the project is to study whether a preparatory online value clarification tool decreases decisional conflict in patients who are considering participation in early phase clinical trials, by improving patient-physician communication with respect to the discussion of patients’ preferences and the decision making process. Following the Medical Research Council – UK – guideline for complex interventions [22], the study consists of two major parts that take place simultaneously, as illustrated in Fig. 1:

1. the development and feasibility testing of the OnVaCT (phase 1 and 2 of MRC framework);
2. the implementation and evaluation of the OnVaCT (phase 3 and 4 of MRC framework).

### Setting

Patients will be included from three Dutch hospitals with sizeable units for early phase clinical research:

- Erasmus MC, Rotterdam;
- Netherlands Cancer Institute – Antoni van Leeuwenhoek hospital, Amsterdam;
- UMC Utrecht.

### Part 1: development and feasibility testing of the OnVaCT

#### Aims

1. To qualitatively study life values of patients facing participation in early phase clinical trials.
2. To design and test an OnVaCT that fits the decision-making processes of patients and working and communication routines of oncologists, thereby supporting patient-physician communication.

#### Study design

The following main phases will be distinguished:

1. development (identifying existing theoretical and empirical evidence; collecting additional evidence; modelling of the tool);
2. feasibility testing/piloting of the modelled OnVaCT.

Information will be gathered prospectively using interviews, focus groups and user sessions with the modelled OnVaCT.

#### Study population

We will include patients with advanced cancer who face the choice whether or not to participate in early phase clinical trials, because standard systemic therapy is not or no longer available for them. Inclusion and exclusion criteria for patients are shown in Table 1. In addition, patient representatives (i.e. an advisory council of patients and relatives) of the Consortium on Palliative Care Southwest Netherlands and healthy subjects (e.g. co-workers) will be involved in the feasibility and pilot testing phase.

**Table 1:** Inclusion and exclusion criteria for patients

Inclusion criteria	Exclusion criteria
* Diagnosed with advanced cancer and eligible for first participation in an early phase clinical trial	* Cognitive impairment (e.g. dementia) according to the medical record
* Aged 18 years or older	
* Sufficient command of the Dutch language	Additional exclusion criteria for part 2:
* Written informed consent	* No access to the Internet
	* Participated in interviews regarding the development of the OnVaCT

To make the OnVaCT meaningful to both patients and medical oncologists, we will also include medical oncologists in the development and pilot testing phase of the study. Medical oncologists and fellow medical oncologists must be a member of the early phase clinical research teams of the participating hospitals to be eligible to participate.

#### Procedures

##### Development phase: clarifying life values through semi-structured interviews

Patients will undergo two serial semi-structured interviews about their life values, which will be (partially) based on a literature search for existing theoretical and empirical evidence. The first interview will take place before the first consult with a medical oncologist about early phase clinical trials. The second interview, which will follow-up on the results from the first interview, will take place approximately 3 weeks after the patient has had their first consultation about early phase clinical trials. Serial interviewing is chosen to neatly fit the evolving and complex decision process of the patient, and to generate “private accounts and descriptions of sensitive topics” that characterize this decision process [23].

In addition, medical oncologists from the participating hospitals will be interviewed via a semi-structured interview about patient-physician interaction, the role of life values in consultation and about their preferences and needs for the OnVaCT to be used in daily work.

After analysing the interviews, there will be four focus groups (i.e. one with patients and three with medical oncologists) to validate the results of the interviews by discussing the main values and themes and requirements and wishes for the OnVaCT that were derived from the interviews. In addition, tool functionalities and tool design will be discussed. Patient representatives will also be involved in the focus groups.

In close collaboration with an ICT company specialized in the development of electronic educational tools and serious games, a theoretical basis for the set-up of the OnVaCT is formulized. The results from the interview and focus groups analyses will be used by the researchers and the ICT company to design the content of the OnVaCT and to further develop the set-up. This will result in a first prototype.

##### Feasibility and pilot testing phase

For feasibility and pilot testing, think aloud sessions with patients, medical oncologists and patient representatives will be organized in which the prototype OnVaCT will be tested and feedback will be registered for further improvement of the OnVaCT. A think aloud session is a method in which subjects perform search, evaluation and application tasks within an online interface while thinking aloud and giving feedback [24]. The think aloud sessions will be individual and the patient sessions will differ and be analysed separately from the oncologists sessions, as the two groups have different needs and interests regarding the OnVaCT.

In this phase, the collaboration with the ICT company will be intensified. To maximize the input of feedback on the OnVaCT functionalities and design, also healthy subjects (e.g. co-workers) will be asked to test the OnVaCT for usability and bug detection on a voluntary base. Based on the results, the OnVaCT will be adapted, and a ready-to-implement OnVaCT will be delivered.

#### Purposeful sampling

Based on research on the calculation of sample sizes for qualitative interviewing [25], purposeful sampling will require a total of 24 serial interviews with 12 patients (or less if saturation – i.e. the interviews no longer provide new insights – is reached). Knowing that a small number of oncologists are collaborating in the three participating early phase clinical research units, we will aim for 2–3 semi-structured interviews per unit (*n* = 6–8 in total).

The total number of patients, representatives and relatives participating in the patient focus group might fluctuate due to the natural courses of the diseases/treatments, but we aim for the focus group to contain 6–8 individuals with a range in patient characteristics. There will be three focus groups with medical oncologists (in training), each in one of the participating hospitals with an early phase clinical research unit and consisting of 3–4 oncologists.

The think aloud sessions for piloting of the value clarification tool will be conducted with patients (*n* = 8–10) and with medical oncologists (*n* = 6). Also healthy subjects and patient representative (s) will be asked to participate in this phase; the number of participants depends on the developmental process. The test sessions with healthy subjects, primarily take place for usability purposes, but may also reveal some additional insights regarding the (presentation of the) content of the tool, since they – like our target patient population – have no prior knowledge about the tool.

#### Qualitative analysis

This study will provide a qualitative thematic analysis [26] of the gathered data. The patient and oncologist interviews will be audio taped and transcribed verbatim, and uploaded to Atlas.ti 8.3.20. A bottom-up approach will be applied that allows for the extraction of general themes and patterns from raw material [27–29]. Focus groups will be both audio- and videotaped and transcribed verbatim. Think aloud sessions will be videotaped for the analysis and summarization of the testing of the content, usability and user experience of the OnVaCT.

The transcripts from the interviews will first be open coded to label data, then axial coded to categorize the labelled data, creating a coding scheme accompanied by memos. The analysis is cyclic, meaning that with every analysis of an interview, the coding scheme with its key concepts, underlying themes, subthemes and explanatory memos will be further completed with the necessary nuances. Analysis of the focus groups will be done in a similar manner as the interviews. Special attention will be paid to the themes and values emerging from the interview study and whether they are recognized by patients participating in the focus group. During the focus groups with medical oncologists, the reaction and mutual interaction about the impact the OnVaCT might have on daily practices will also receive special attention. Lastly, think aloud sessions will be analysed to assess usability and perceived usefulness [24]. For the assessment of usability, we will especially focus on the person’s ability to navigate and find the desired information and his ideas on the layout. Perceived usefulness is about the content presented in the OnVaCT in terms of comprehension, presentation of the content, and the applicability of the content to the patient.

### Part 2: implementation and evaluation of the OnVaCT

#### Aims

1. To evaluate the effect of implementation of the OnVaCT with accompanying training on:
  - patients’ decisional conflict (primary outcome);
  - patient-physician communication regarding the discussion of patients’ preferences and the decision making process (secondary outcome).
2. To investigate the actual usage of the OnVaCT (secondary outcome).

#### Study design

To evaluate the OnVaCT, we will perform a prospective pre-test post-test multi-centre clinical study to investigate the effect of implementation of the OnVaCT (Fig. 1). In the period between pre-test and post-test the OnVaCT will be implemented. Results on decisional conflict and patient-physician communication measured during the first period of 12–18 months before implementation of the OnVaCT (pre-test) will be compared with the results measured during a second period of 12–18 months after implementation (post-test). In both the pre-test and the post-test data will be collected at three time points:

1. before the actual visit to the unit for early phase clinical research: registration of baseline characteristics;
2. the initial visit with a medical oncologist regarding early phase clinical studies: measurement of patient-physician communication;
3. three weeks after T2: measurement of decisional conflict.

#### Study population

Similar to part 1, we will include patients with advanced cancer who face the choice whether or not to participate in early phase clinical trials, because standard systemic therapy is not or no longer available for them (Table 1). Additionally, patients need to have access to the Internet and patients included in part 1 are excluded for part 2 of the project.

#### Procedures

##### Implementation phase

When the members of the project team agree on the content of the OnVaCT, we will develop a training session for the medical oncologists of the early phase clinical research units. All members of the study teams will be informed about the forthcoming implementation of the tool and trained how to handle the preparatory work of the patient by using the tool and how to integrate patients’ values and preferences and further information needs in the communication with patients facing a choice whether or not to participate in an early phase clinical trial. We will use the barriers as perceived by caregivers of the team as explored during the development of the OnVaCT as input for the development of the accompanying training module. The module will be developed in close collaboration with a psychologist with educational experience, and based on previously developed training modules in e.g. the VOICE study, in which clinicians were trained in exploring patients’ preferences and how to use a preparatory tool during consultations [30], and the CHOICE trial, in which a training was developed on shared decision making in consultations about palliative care, specifically for oncologists [31].

Directly after the training, the OnVaCT will be implemented in clinical practice, and the post-test will start. Patients in the post-test will use the tool in preparation of their first visit to a medical oncologists regarding early phase clinical trials.

##### Evaluation phase (pre-test and post-test)

After giving preliminary oral consent for participation in this study, patients will receive a link to the first questionnaire (T1) via e-mail, which they have to complete before the initial visit with the medical oncologist (T2). The questionnaire starts by asking if the patient gives his/her consent to participate in that particular questionnaire. In the post-test, an additional question will ask whether the patient understands and agrees that data from the OnVaCT will be shared with the oncologist with whom he/she has an appointment. After finishing the first questions, patients participating in the post-test will receive a direct link to the OnVaCT and additional questions regarding technology acceptance and satisfaction with the tool. During both the pre-test and the post-test, written informed consents will be signed and/or collected immediately before the initial visit with the medical oncologist (T2). Subsequently, the initial consultation itself (T2) will be (video- and) audiotaped. Decisional conflict will be measured by means of a final questionnaire 3 weeks after the initial consult with a study medical oncologist (T3).

#### Measurements

Table 2 gives an overview of the baseline and outcome measurements in part 2 of the OnVaCT project.

**Table 2:** Measurements in part 2 of the OnVaCT project

Outcome	Instrument	Source
Baseline measurements		
Patient’s health literacy	Set of Brief Screening Questions (SBSQ-D): Dutch version [32, 33] of the 3-item health literacy scale of Chew et al. [30] on 5-point Likert scales.	Questionnaire at T1
Sense of hope	Herth Hope Index (HHI) [34, 35]: Items on a 4-point Likert scale on three dimensions: temporality and future, positive readiness and expectancy, and interconnectedness.	Questionnaire at T1
Technology acceptance	Measurements from the Technology Acceptance Model (TAM), adapted in the Unified Theory of Acceptance and Use of Technology (UTAUT) [36–38]. In total, 15 items on a 7-point Likert scale are included.	Questionnaire at T1, only in post-test
Satisfaction with the tool	Website Satisfaction Scale [39–42]: A Dutch version of the Website Satisfaction Scale will be used, consisting of the subscales ‘satisfaction with comprehensibility’ (3 items), ‘satisfaction with attractiveness’ (5 items) and ‘satisfaction with emotional support’ (4 items), all on a 7-point Likert scale.	Questionnaire at T1, only in post-test
Quality of life	QLQ-C30 version 3.0 [43] of the European Organisation for Research and Treatment of Cancer (EORTC): 28 items on a 4-point Likert scale and 2 items on a 7-point Likert scale.	Questionnaire at T1
Primary outcome		
Decisional conflict	Decisional Conflict Scale (DCS) [25]: 16 items on a 5-point Likert scale from 0 to 4. The items are summed, divided by 16 and multiplied by 25 to get a total score for decisional conflict on a 0–100 scale.	Questionnaire at T3
Secondary outcomes		
Extent to which caregivers involve patients in shared decision-making	Adapted Observer OPTION^MCC^ [44] based on the OPTION^5^ [45]: in each recorded consultation, 7 behavioural competences for the medical oncologists regarding goal talk (1 item), option talk (2 items), team talk (1 item), decision talk (2 items) and evaluation talk (1 item) will be coded on a 5-point Likert Scale (0 = ‘the behaviour is not observed’ – 4 = ‘the behaviour is observed and executed to a high standard’). Following OPTION^5^ guidelines, these scores will be transformed to a 0–100 score.	Analysis of recorded consultation
Discussion of patient preferences and values	All values and preferences discussed during the consultation will be coded by using a codebook that will be developed specifically for this study, distinguishing between the contribution of the patient, relative (s) and caregiver. This codebook will be (partly) based on the values and preferences that are distinguished in part 1 of the OnVaCT-project.	Analysis of recorded consultation
Duration of the consultation	The length of the consultation will be assessed by measuring the length of the recorded consultation in minutes.	Analysis of recorded consultation
Actual usage of the tool	To analyse the actual usage of the tool, Google Analytics will be used to log the number of website visits (i.e. the number of times someone visited/used the tool), the time spent on the website (i.e. the accumulated time someone used the tool) and the number and kind of pages viewed.	Tracking data at T1, only in post-test

##### Baseline measurements

The background questionnaire for the evaluation of the OnVaCT contains socio-demographic items on age, gender, education, living situation, computer experience and online-surfing behaviour. Medical background characteristics (e.g. diagnosis, time since diagnosis, health status, WHO performance status) will be collected from the medical file. In addition, several potential confounding factors will be measured (see also Table 2):

- patients’ health literacy;
- sense of hope;
- technology acceptance (only in post-test);
- satisfaction with the tool (only in post-test);
- quality of life.

##### Outcome measurements

The primary outcome (Table 2) is decisional conflict, i.e. the extent to which patients feel insecure about their decision regarding participation in early phase clinical trials. Secondary outcomes (Table 2) are the communication process (consisting of the extent to which caregivers involve patients in shared decision-making; the discussion of patients’ values and preferences; and the consultation duration) and the actual usage of the OnVaCT (which will be tracked by using Google Analytics).

#### Sample size calculation

The sample size calculation is based on decisional conflict as the primary outcome. Following literature, we assume a mean decisional conflict score of 27 (sd = 12) in the pre-implementation period [7]. To acquire an 80% power to detect an effect size of .30 with an alpha-level of .05 (one-sided), a total sample size of 276 patients is required (138 in the pre-test before the implementation of the OnVaCT and another 138 after the implementation). The three participating hospitals currently see approximately 400–500 new patients a year who consider participation in phase I clinical trials. Based on previous research, we expect a response rate of approximately 40%. We thus need approximately 12 months in the pre-test and another 12 months in the post-test to recruit the required number of patients. We will start the pre-test 18 months before the implementation of the OnVaCT, and if needed, we can spend another 18 months for the post-test. Hence, the inclusion of 276 participants based on the sample size calculation is feasible.

#### Statistical analysis

All analyses regarding the evaluation of the OnVaCT will be performed in the most recent version of IBM SSPS Statistics. Descriptive statistics and frequency distributions will be generated for the patients’ demographics and medical disease characteristics. For the analysis of the main and secondary outcome measures, an intention-to-treat analysis will be used, thereby including patients who were included in the post-test group (i.e. after the implementation of the OnVaCT with accompanying training) regardless of whether or not they used the OnVaCT.

For the analysis of decisional conflict, a one-sided t-test will be performed between the pre- and post-test measurements. Patient demographics, performance status, health literacy and the level hope may be used as covariates to correct for possible imbalance between the patients from the two study periods (pre-test and post-test) by means of regression analysis. In addition, a mediation analysis will be performed using Structural Equation Modelling (SEM) to calculate the direct effect of the use of the OnVaCT on decisional conflict and to what extent that effect was mediated by communication during the consultation (i.e. the extent to which patients are involved in the decision making process as measured with the adapted Observer OPTION^MCC^ [44]; and the extent to which patient values and preferences are discussed as measured with a codebook we will develop). In the SEM analysis, patient demographics, performance status, health literacy, the sense of hope and the quality of life at baseline may be used as covariates to correct for possible imbalance between the patients from the two study periods (pre- and post-test).

Secondary outcomes, e.g. the total score for the shared decision making process during consultation, the discussion of patients’ values and preferences by patients, relatives and caregivers and the length of the consultation will be compared between the two test periods using two-sided t-tests. Again, the regression analysis may be used to correct for possible differences in patient demographics, performance status, health literacy and hope between the patients from the two study periods (pre- and post-test). Descriptive statistics will be generated for technology acceptance and satisfaction with the tool, which are only measured in the post-test.

## Discussion

The importance of incorporating patient values in patient-physician communication has received increased attention recently. Over the years, value clarification methods or exercises have been developed as part of a decision aid [46]. The OnVaCT project however is developed from a different perspective, using digital communication (serious gaming), to help patients articulate their values and perspectives related to their decisions for participation in an early phase clinical trial and/or a palliative care trajectory. By using the OnVaCT before the appointment with a medical oncologist of an early phase clinical research unit, patients are assumed to share and formulate their personal values with their oncologist more easily, which in turn may support oncologists in tailoring the information to the patients’ needs. We aim to develop the OnVaCT as a comprehensive package with a training module for oncologists to assist them in discussing patients’ results of the OnVaCT. As the decisional conflict scale appears viable for measuring the quality of end-of-life decision making [47], a decrease in decisional conflict can be seen as an improvement of patients’ decision making process, which may contribute to an improved quality of life [8, 9].

We will use decisional conflict as our primary endpoint, which is the most frequently used scale in studies aimed at improving medical decision making [48]. For this endpoint, the timing of measurements is essential. However, a scoping review revealed that thus far only few studies have measured both the decision-making stage and decisional conflict [49]. We have chosen to measure decisional conflict 3 weeks after a patient’s initial consultation with a medical oncologist. Patients often have a (telephone) appointment with the medical oncologist or a nurse practitioner about 1 week after the initial consultation to ask for or confirm their decision regarding trial participation. The 3 week time frame therefore offers most patients sufficient opportunity to make a decision and to let that decision sink in, but is, obviously, too early for patients to have already started treatment in an early phase clinical trial. By analysing the communication process in the first consultation that patients have with the medical oncologist regarding participation in such a trial and incorporating this into SEM analysis, this is one of the first studies that is able to link the process during the consultation to decisional conflict.

A longitudinal follow-up study is necessary to evaluate the potential effects of the OnVaCT on a longer term than 3 weeks. Additionally, it seems important to integrate patient-reported preferences along the entire cancer trajectory, as patient preferences may change in relation to treatment experiences, coupled with the impact on quality of life [17, 50]. A longitudinal study could therefore also reveal the effect of the experienced burden of trial participation, (potentially) exhausting logistics or, ultimately, profiting from participation in a trial or not on decisional conflict. Since the current study includes patients with advanced cancer who are considering participation in early phase clinical trials, both patients who eventually chose to participate in an early phase clinical study and those who chose not to participate will be included.

The OnVaCT study uses both qualitative and quantitative research methods to develop and perform the initial testing phase for a value clarification tool that is meaningful for both patients and oncologists. This mixed-methods approach offers the opportunity to build on in-depth analyses to gain a better understanding of the underlying processes of difficult and potentially life-changing choices to create an OnVaCT that suits patients’ decision making processes, but also to quantitatively evaluate the OnVaCT. The OnVaCT (with training module) will already be implemented in clinical practice during the evaluation of the tool in the three largest centres for early phase clinical research in the Netherlands (located in Rotterdam, Utrecht, and Amsterdam), making it easy to continue using the OnVaCT when it appears to be of value during the evaluation. Additionally, studies regarding other (end-of-life) decisions, which may pose a similar type of decision conflict, may also benefit from the results of the current project. Even though the benefit from treatment may be known, and therefore more precise arguments pro and contra treatment can be made, patients who are able to identify and realize their personal values regarding these treatments, could still benefit from less decisional conflict and more attuned discussions with their oncologists regarding the choices they make.

If this project has its hypothesized positive outcome, meaning that decisional conflict indeed seems to decrease by using the OnVaCT, the project can be followed up in different manners. For instance, regional hospitals could be asked to participate and use the OnVaCT to aid in decision making after “bad news” has been delivered on the absence of regular treatment options, which could help improve specific referral for early phase clinical trials. As the use of the OnVaCT in peripheral hospitals provides all eligible patients to consider their preferences regarding participation in early phase clinical trials, patients who come to the conclusion that participation is not suitable for them, do not have to be referred to an early phase clinical research unit at all. As only a specific selection of patients who actually consider participating in early phase clinical trials will be referred, the medical oncologists could perhaps attune their consultations even better at this particular population.

## Challenges and limitations

The main challenge of this project concerns the feasibility of timely reaching, informing, and inclusion of patients willing to participate in the first interview or the first questionnaire. Referral time to an early phase clinical trial centre is usually short (approximately 1 week). It is critical that there is close contact between the clinical trial managers and the executing researchers (NS and LL), so that patients can be timely approached for planning and conducting an interview or completion of the first questionnaire before the initial consult about potential participation in an early phase clinical trial takes place.

A first limitation of the current project is that although our hypothesis that using the OnVaCT will decrease decisional conflict, it is also possible that the opposite will be the case. After all, when patients are asked to use the OnVaCT, we may make them more aware of the choices they have towards the end of their lives, which in turn may cause them to think more about these choices and lead to a different decision or increase their experienced decisional conflict as a result. Additionally, all oncologists whose consultations will be recorded, will be asked for consent to make these recordings and may consequently be made more aware of what and how they communicate with their patients. This could mean that the oncologists possibly already focus more on patient values during the pre-test than they would do otherwise, which may cause a bias in our pre-test. Nevertheless, being aware that patient values should play a role in the consultations does not necessarily mean that the consultations cannot still be improved or personalized.

It is also important to realize that the pre-test post-test study design could also cause another potential bias in the current project due to the rapidly evolving cancer drug developments, and patients’ perceptions about these (new) drugs as a result. Previous studies have shown multiple shifts in phase I oncology trials over (more than) a decade, including (but not limited to) changes in the distribution of cancer types among patients who participate in these trials [51], patient characteristics and trial designs [52], and decreased toxic death rates [53]. Since the start of the pre-test and the end of the post-test are about 2.5 years apart (see Fig. 1), it may be assumed that shifts in phase I oncology trials are less significant over the inclusion periods of the present study.

## Concluding remark

In short, this project will generate an OnVaCT that aims to assist patients with advanced cancer for whom standard treatment options are exhausted to clarify their values facing a difficult end-of-life decision, e.g. the participation in an early phase clinical trial. The OnVaCT may thus help these patients to better determine their preferences regarding experimental treatment. By sharing these results with the medical oncologist, the communication during a consultation may be better attuned to individual patient’s needs and thereby support him or her in the decision whether or not to participate in an early phase clinical trial, probably resulting in less decisional conflict.

## Data Availability

Not applicable.
